# Mechanisms of ferroptosis and targeted therapeutic approaches in urological malignancies

**DOI:** 10.1038/s41420-024-02195-w

**Published:** 2024-10-09

**Authors:** Wenjie Ma, Xiaotian Jiang, Ruipeng Jia, Yang Li

**Affiliations:** Department of Urology, Nanjing First Hospital, Nanjing Medical University, Nanjing, Jiangsu People’s Republic of China

**Keywords:** Urological cancer, Cell biology

## Abstract

The prevalence of urological malignancies remains a significant global health concern, particularly given the challenging prognosis for patients in advanced disease stages. Consequently, there is a pressing need to explore the molecular mechanisms that regulate the development of urological malignancies to discover novel breakthroughs in diagnosis and treatment. Ferroptosis, characterized by iron-ion-dependent lipid peroxidation, is a form of programmed cell death (PCD) distinct from apoptosis, autophagy, and necrosis. Notably, lipid, iron, and glutathione metabolism intricately regulate intracellular ferroptosis, playing essential roles in the progression of various neoplasms and drug resistance. In recent years, ferroptosis has been found to be closely related to urological malignancies. This paper provides an overview of the involvement of ferroptosis in the pathogenesis and progression of urological malignancies, elucidates the molecular mechanisms governing its regulation, and synthesizes recent breakthroughs in diagnosing and treating these malignancies. We aim to provide a new direction for the clinical treatment of urological malignancies.

## Facts

- Ferroptosis plays a crucial role in the progression and drug resistance of various tumors by modulating lipid, iron, and glutathione metabolism.
- Ferroptosis is closely associated with the pathogenesis and progression of urologic malignancies.
- Ferroptosis may represent a novel therapeutic direction for the treatment of urologic malignancies.

## Open questions

- What mechanisms underlie the promotion of urologic malignancies and drug resistance by ferroptosis?
- Have reliable markers of ferroptosis in urologic malignancies been identified?
- What strategies can be employed to target ferroptosis to enhance therapeutic efficacy against urologic malignancies?

## Introduction

According to GLOBOCAN 2020, patients with urinary malignancies constitute approximately 13% of the total number of cancer patients. The top three cancers in this category are prostate cancer (PCa), bladder cancer (BCa), and renal cell carcinoma (RCC) [1]. Despite advancements in diagnostic and therapeutic methods for urologic tumors that have led to notable enhancements in patient prognosis [2, 3], the mortality rate for individuals in the advanced stages of genitourinary cancer remains high [4]. This imposes a considerable burden on public health services and necessitates ongoing efforts to address the challenges associated with these conditions [4].

In multicellular organisms, regulatory cell death (RCD) is an essential homeostatic mechanism that maintains tissue morphology and function. Ferroptosis is a distinctive form of iron-dependent RCD that differs from apoptosis, necrosis, and autophagy in terms of cell morphology and biochemical characteristics [5]. Ferroptosis is intricately mediated by the interplay of iron metabolism, lipid metabolism, and glutathione (GSH) metabolism and involves a complex network of interactions within diverse metabolic pathways [6]. Ferroptosis, which is characterized by iron-mediated oxidative damage, is triggered by the high expression of unsaturated fatty acids on the cell membrane. This iron-dependent process results in lipid peroxidation, ultimately leading to cell death [7].

Increasing evidence suggests that a deeper understanding of the potential molecular mechanisms of urinary malignancies may be a promising way to enhance treatment strategies and improve prognostic outcomes [8, 9]. Recently, an increasing number of studies have shown that ferroptosis may also participates in the pathophysiological processes of malignancies of the urinary system and has attracted increasing attention from urologists. Therefore, this review aims to summarize current studies regarding the impact of ferroptosis on urinary system malignancies.

## Mechanisms of ferroptosis

**Fig. 1:**
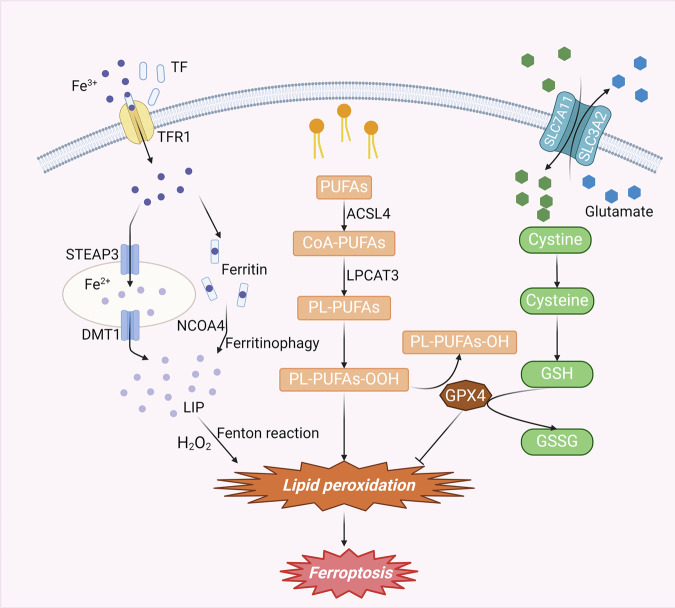
Mechanisms of ferroptosis. The conversion of PUFAs to PL-PUFAs can be mediated by *ACSL4* and *LPCAT3*. PL-PUFAs can be easily converted to PL-PUFA-OOH through various pathways, which can cause lipid peroxidation and ferroptosis. Extracellular Fe^3+^ enters the cell by binding to TF and undergoes reduction or ferritinophagy, ultimately converting into Fe^2+^ and forming the *LIP*. When *LIP* becomes overloaded with iron, it becomes prone to a Fenton reaction, facilitating lipid peroxidation and thereby triggering ferroptosis. The cysteine transported into cells by *SLC7A11* can be converted into cystine, which is an essential component of GSH. GSH, mediated by *GPX4*, can be transformed into *GSSG* while reducing PL-PUFA-OOH to PL-PUFA-OH, thereby inhibiting ferroptosis. Abbreviations: PUFAs, polyunsaturated fatty acids; CoA, coenzyme A; PL-PUFAs, phospholipid-containing PUFAs; *ACSL4*, acyl-CoA synthetase long-chain family member 4; *LPCAT3*, lysophosphatidylcholine acyltransferase 3; *TF*, transferrin; *TFR1*, transferrin receptor 1; *STEAP3*, iron oxide reductase steam 3; *DMT1*, divalent metal transporter 1; *NCOA4*, nuclear receptor coactivator 4; *LIP*, labile iron pool; *SLC7A11*, solute carrier family 7 member 11; *SLC3A2*, solute carrier family 3 member 2; GSH, glutathione; *GSSG*, Oxidized glutathione; *GPX4*, glutathione peroxidase 4.

Ferroptosis is a multifaceted biological process primarily instigated by the dysregulated metabolism of iron, GSH, and polyunsaturated fatty acids (PUFAs) (Fig. 1).

### Lipid metabolism

Lipid peroxidation and iron metabolism are pivotal biochemical processes that culminate in ferroptosis [10]. Moreover, lipid peroxidation is considered a marker of ferroptosis [11]. PUFAs in the body are metabolized by acyl-coenzyme A (*CoA*) synthetase long-chain family member 4 (*ACSL4*) into CoA-PUFAs, which subsequently undergo conversion to phospholipid-containing PUFAs (PL-PUFAs) mediated by lysophosphatidylcholine acyltransferase 3 (*LPCAT3*) [12]. Due to the dienyl groups in PUFAs, PL-PUFAs are susceptible to the generation of PL-PUFA-OOH through peroxidation catalyzed by iron ions via the Fenton reaction [13, 14]. Additionally, oxidation by lipid oxidases (*ALOXs*) or cytochrome P450 oxidoreductases (*PORs*) can also result in the production of substantial quantities of peroxidized phospholipids (PLOOHs) and breakdown products of lipid peroxidation [15, 16]. These products contribute to the disruption of cell membrane integrity, ultimately leading to cell death.

### Iron metabolism

Iron plays a pivotal role in various metabolic pathways in the human body, contributing significantly to the regulation of numerous cellular biological functions [17]. Typically, iron ions in the circulation are present in the form of Fe^3+^. When extracellular Fe^3+^ binds to transferrin (*TF*), it is recognized by transferrin receptor 1 (*TFR1*) on the cell membrane, facilitating its transportation into the cell. The reduction of Fe3+ to Fe^2+^ in the endosome is mediated by iron oxide reductase steam 3 (*STEAP3*). Subsequently, divalent metal transporter 1 (*DMT1*) facilitates the transport of divalent iron into the cytoplasm, where it forms a labile iron pool (*LIP*). Excess iron will be present in the form of ferritin. Therefore, under normal physiological conditions, iron in the body typically exists in the form of Fe^2+^, Fe^3+^ and ferritin and maintains metabolic homeostasis [18]. When iron metabolism is disrupted by the activation of nuclear receptor coactivator 4 (*NCOA4*)-mediated ferritinophagy for various reasons, intracellular Fe^2+^ reaches an overloaded state [19]. Fe^2+^, which is unstable, readily engages in a Fenton reaction with hydrogen peroxide. This interaction leads to the production of hydroxyl radicals, which possess potent oxidizing properties, and subsequently generates substantial amounts of lipid peroxides [20]. Moreover, excess iron augments reactive oxygen species (ROS) production by activating ROS-generating enzymes, including nicotinamide adenine dinucleotide phosphate oxidase and lipoxygenase [21, 22]. The excessive accumulation of ROS and lipid peroxidation ultimately results in ferroptosis.

### GPX4/GSH homeostatic system

The system *Xc-/GSH/GPX4* regulatory axis is the primary intracellular defense mechanism against the initiation of ferroptosis [23]. System Xc-, a member of the heterodimeric amino acid transporter protein family, comprises solute carrier family 3 member 2 (*SLC3A2*) and solute carrier family 7 member 11 (*SLC7A11*), which are linked by disulfide bonds within the cellular membrane. Notably, *SLC7A11* serves as the major functional subunit in system Xc-. This system facilitates the transport of cysteine into the cell while simultaneously exporting glutamate out of the cell at a 1:1 ratio [24, 25]. Cystine is reduced to cysteine upon entry into the cell, which is involved in the composition of GSH. *GPX4*, the exclusive reductive enzyme in the GPX family, plays a pivotal role in reducing phospholipid hydroperoxides (LOOHs) to nontoxic lipohydrols (LOHs), effectively inhibiting iron death. The concerted action of GPX4 with the cofactor GSH is instrumental in mitigating oxidative stress by depleting lipid peroxides and neutralizing ROS [26]. Inhibition of system Xc impedes intracellular GSH biosynthesis, thereby compromising the lipid membrane repair capacity of *GPX4*. This disruption increases the accumulation of toxic lipid free radicals and ROS, fostering lipid peroxidation and ultimately culminating in ferroptosis [27, 28].

## The molecular mechanism of ferroptosis in urological malignancies

Recent studies have demonstrated that ferroptosis may be involved in the initiation of urological malignancies and can contribute to the progression of urological malignancies via distinct mechanisms (Table 1).

**Table 1:** The molecular mechanism of ferroptosis in urological malignancies.

Cancer	Drug	Target	Ferroptosis (inducer inhibitor)	Mechanism	Model	Reference
RCC		KLF2	Induce	Bind with the GPX4 promoter region	In vitro and in vivo	[29]
	Salinomycin	PDIA4	Induce	PERK/ATF4/SLC7A11 signaling pathway	In vitro and in vivo	[30]
		lncRNA SLC16A1-AS1	Inhibit	miR-143-3p ↓ /SLC7A11 signaling↑	In vitro and in vivo	[31]
	Icariside II		Induce	miR-324-3p/GPX4 axis	In vitro and in vivo	[32]
		KLF11	Induce	Downregulate the protein expression of ferritin, system xc (-) cystine/glutamate antiporter (xCT), and GPX4	In vitro and in vivo	[33]
		ISCA2-deficient	Induce	Trigger the iron starvation response, resulting in iron/metals overload	In vitro and in vivo	[35]
		SUV39H1	Inhibit	Suppress iron accumulation and lipid peroxidation, inhibit the expression of DPP4	In vitro and in vivo	[36]
	Cyst(e)inase-rapamycin		Induce	Deplete the exogenous cysteine/cystine supply, promote ferritins’ destruction	In vitro and in vivo	[37]
		AIM2	Inhibit	FOXO3a ↑ -ACSL4↓ axis	In vitro and in vivo	[39]
	Adipokine chemerin		Induce	Suppresses fatty acid oxidation	In vitro and in vivo	[41]
		ACSL3	Induce	Rely on access to exogenous fatty acids to drive lipid droplet deposition	In vitro and in vivo	[42]
		MLYCD	Induce	Inhibit fatty acid synthesis and lipid droplet accumulation	In vitro and in vivo	[43]
		dipeptidyl peptidase 9 (DPP9)	Inhibit	KEAP1-NRF2-SLC7A11 axis	In vitro and in vivo	[45]
	Disulfiram/copper		Inhibit	Prolong the half-life of NRF2 and reduced its degradation, reduce the expression of NPL4	In vitro and in vivo	[46]
		OGT	Induce	Inhibit the degradation of HIF-2a	In vitro and in vivo	[48]
		SDH inhibition	Induce	Eliminate mitochondrial ROS levels, decrease cellular ROS and diminish peroxide accumulation	In vitro	[49]
PCa	low-dose antimony		Inhibit	Active of the Nrf2-SLC7A11-GPX4 pathway	In vitro and in vivo	[50]
	Flubendazole		Induce	Induce the expression of P53, suppress SLC7A11/GPX4	In vitro and in vivo	[51]
		lncRNA OIP5-AS1	Inhibit	miR-128-3p/SLC7A11 signaling	In vitro and in vivo	[52]
		CEMIP	Inhibit	ITPR3/CaMKII/NRF2/SLC7A11 pathway	In vitro and in vivo	[54]
		HnRNP L knockdown	Induce	Produce more IFN-γ to induce the ferroptosis in CRPC cells via STAT1/SLC7A11/GPX4 signaling axis	In vitro and in vivo	[55]
		PHGDH	Inhibit	Increase GSH/GSSG levels and decrease LipROS production as well as promote SLC7A11 expression through suppression of the p53 signaling pathway	In vitro and in vivo	[56]
		SGK2	Inhibit	Induce the translocation of FOXO1 from the nucleus to the cytoplasm, relieving the inhibitory effect of FOXO1 on GPX4	In vitro and in vivo	[57]
	Evoldiine		Induce	Decrease the expression of GPX4	In vitro and in vivo	[58]
	TQB3720		Induce	Reduce the AR/SP1 transcriptional complex binding to GPX4 promoter	In vitro and in vivo	[59]
	PMANs		Induce	Increase GSH consumption, suppressed SLC7A11 and GPX4 expression, and promoted the generation of ROS and LPO	In vitro and in vivo	[60]
		RB1-deficient	Induce	RB ↓ /E2F ↓ /ACSL4↑ molecular axis	In vitro and in vivo	[61]
	Enzalutamide		Induce	Decrease GSH production, increased lipid peroxidation	In vitro and in vivo	[62]
		DECR1	Inhibit	Inhibit cellular accumulation of PUFAs, suppress mitochondrial oxidative stress and lipid peroxidation	In vitro and in vivo	[63]
	Polyphyllin I (PPI)		Induce	ERK/DNMT1/ACSL4 axis	In vitro and in vivo	[64]
	SupraT		Induce	Ferritinophagy	In vitro and in vivo	[65]
	Luteolin		induce	Promote TFEB nuclear translocation and increase ferritinophagy	In vitro and in vivo	[66]
		AOC1	Induce	SOX15/AOC1/ROS axis	In vitro and in vivo	[67]
Bca	NCT-502	PHGDH	Inhibit	PCBP2 ↑ / SLC7A11↑	In vitro and in vivo	[69]
		EMP1	Induce	SLC7A11↓	In vitro and in vivo	[70]
	Fin56		Induce	Autophagy-mediated GPX4 degradation	In vitro	[71]
		p53	Induce	Activate the lipoxygenase activity of ALOX15B via inhibiting SLC7A11	In vitro and in vivo	[72]
		HSPA5	Inhibit	P53/SLC7A11/GPX4	In vitro and in vivo	[73]
	BQR@MLipo		Induce	Induce extensive mitochondrial lipid peroxidation and ROS	In vitro and in vivo	[74]
		FLRT2	Induce	Elevate ACSL4 expression	In vitro	[76]
		PCBP1	Inhibit	LACTB ↓ /PISD↑	In vitro and in vivo	[77]
	Erianin	NRF2↓	Induce	Promote the accumulation of lethal lipid-based ROS and the depletion of GSH	In vitro and in vivo	[79]
		WTAP	Inhibit	Install its methylation to 3’-UTR of endogenous antioxidant factor NRF2 RNA enhanced mRNA stability of NRF2	In vitro and in vivo	[80]

### Molecular mechanism of ferroptosis in RCC

**Fig. 2:**
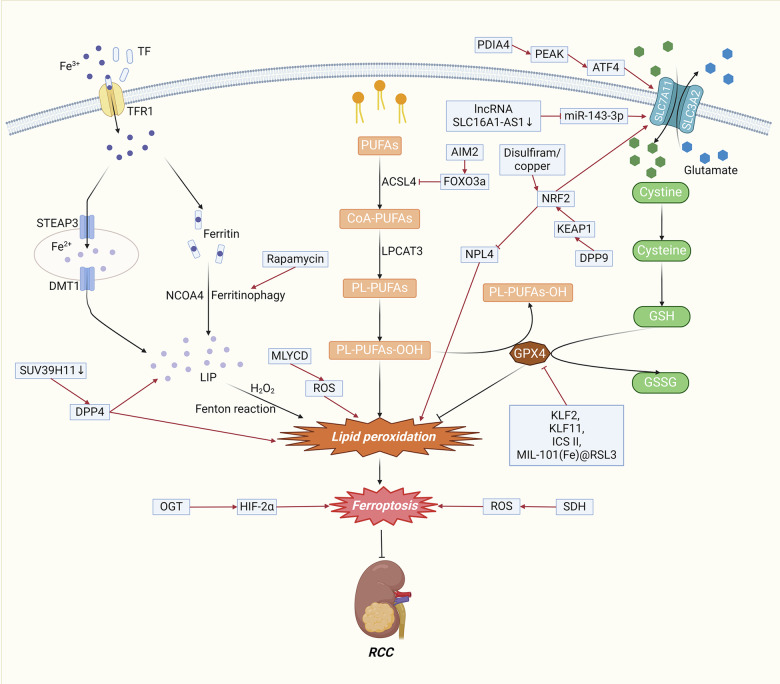
The molecular mechanism of ferroptosis in RCC. The modulation of ferroptosis can exert significant effects on RCC through various mechanisms, encompassing the regulation of GSH levels, lipid metabolism, iron metabolism, the *NRF2* signaling pathway, the *HIF-2α* signaling pathway, and the ROS signaling pathway. Abbreviations: PUFAs, polyunsaturated fatty acids; CoA, coenzyme A; PL-PUFAs, phospholipid-containing PUFAs; *ACSL4*, acyl-CoA synthetase long-chain family member 4; *LPCAT3*, lysophosphatidylcholine acyltransferase 3; *TF*, transferrin; *TFR1*, transferrin receptor 1; *STEAP3*, iron oxide reductase steam 3; *DMT1*, divalent metal transporter 1; *NCOA4*, nuclear receptor coactivator 4; *LIP*, labile iron pool; *SLC7A11*, solute carrier family 7 member 11; *SLC3A2*, solute carrier family 3 member 2; *GSH*, glutathione; *GSSG*, Oxidized glutathione; *GPX4*, glutathione peroxidase 4; *KLF*, Kruppel-like factor; *ICS II*, Icariside II; *DPP4*, dipeptidyl-peptidase-4; *NRF2*, nuclear factor erythroid 2-related factor 2; *OGT*, O-GlcNAc transferase; *HIF-2α*, hypoxia-inducible factor-2α; *SDH*, succinate dehydrogenase; ROS, Reactive Oxygen Species.

The regulation of ferroptosis can impact RCC through multiple mechanisms, including the modulation of GSH levels, lipid metabolism, iron metabolism, the *NRF2* signaling pathway, and the *HIF-2α* signaling pathway (Fig. 2).

#### *GPX4/GSH* steady-state system

GSH-related metabolism significantly affects the activity of the system *Xc-/GPX4* antioxidant axis and consequently the sensitivity of cells to ferroptosis. Lu et al. reported that one of the transcription factor families, Kruppel-like Factor 2 (*KLF2*), is sufficient to induce ferroptosis by suppressing the transcriptional repression of *GPX4*. The decreased expression of *KLF2* detected through immunohistochemistry in primary metastatic ccRCC was strongly associated with poor clinical outcomes [29]. *SLC7A11* can promote GSH synthesis and GPX4 activity and inhibit ferroptosis in RCC cells [21]. Kang et al. identified a positive correlation between *PDIA4* and the *PERK/ATF4/SLC7A11* signaling pathway through bioinformatics analyses of clinical RCC samples and databases [30]. Li et al. reported that the inhibition of the long noncoding RNA (lncRNA) *SLC16A1-AS1* could result in ferroptosis through the *miR-143-3p/SLC7A11* signaling pathway in RCC [31]. Yu et al. reported that Icariside II (ICS II) induces ferroptosis in RCC cells by modulating the *miR-324-3p/GPX4* axis, which is characterized by the accumulation of Fe^2+^, MDA and ROS and a reduction in GSH levels [32]. Zhou et al. reported that KLF11 induced ferroptosis by downregulating the protein expression of GPX4, ferritin, and system Xc-cystine/glutamate antiporter (xCT) [33].

#### Iron metabolism

Increased intracellular free iron leads to ferroptosis, which can be regulated by regulating intracellular free iron levels through iron uptake, utilization, storage, and translocation. The VHL gene is the most commonly mutated gene in RCC. Inactivation of the *VHL* gene leads to downstream accumulation of hypoxia-inducible factor (HIF)-1α and *HIF-2α* [34]. Green et al. discovered that the production of *HIF-1α* and *HIF-2α* can be inhibited by targeting iron sulfur cluster assembly 2 (*ISCA2*). Mechanistically, the inhibition of *ISCA2* initiates the iron starvation response, leading to overload. of iron and other metals, ultimately resulting in ferroptosis [35]. *SUV39H1*, encoding a histone H3 lysine 9 methyltransferase, is frequently upregulated in ccRCC tumors. Wang et al. reported that decreasing the expression of *SUV39H1* could induce iron accumulation and lipid peroxidation, leading to ferroptosis that inhibits ccRCC cell growth. They also reported that *SUV39H1* deficiency resulted in the upregulation of dipeptidyl-peptidase-4 (*DPP4*), which leads to ferroptosis [36]. For hereditary leiomyomatosis and renal cell cancer (HLRCC), Kerimoglu et al. showed that the combination of cyst(e)inase and rapamycin could induce ferroptosis. Mechanistically, cyst(e)inase induces ferroptosis through depletion of the exogenous cysteine/cystine supply, while rapamycin diminishes cellular ferritin levels by facilitating the degradation of ferritins through ferritinophagy [37].

#### Lipid metabolism

Lipid peroxidation is considered a marker of ferroptosis. The absent in melanoma 2 (*AIM2*) protein complex plays a crucial role in the pathogenesis of RCC through its dysregulated expression and activation [38]. Wang et al. reported that *AIM2* promoted *FOXO3a* phosphorylation and proteasome degradation, thereby reducing its transcriptional effect on *ACSL4* and inhibiting ferroptosis [39]. *ACSL4* and *LPCAT3* are involved in the conversion of PUFAs into PUFA-PLs, which in turn are involved in the process of lipid peroxidation [40]. Using a multiomics approach, Tan et al. revealed that chemerin restrained fatty acid oxidation, thus inhibiting ferroptosis [41]. Klasson et al. reported that acyl-CoA synthetase 3 (*ACSL3*) enhances ferroptosis sensitivity through the composition of exogenous fatty acids to drive lipid droplet deposition [42]. Zhou et al. reported that, mediated by malonyl-CoA decarboxylase (*MLYCD*)-facilitated fatty acid oxidation, inhibition of lipid droplet accumulation disrupted endoplasmic reticulum and mitochondrial homeostasis and increased ROS levels, ultimately culminating in the induction of ferroptosis [43].

#### *NRF2* signaling pathway

The transcription factor nuclear factor erythroid 2-related Factor 2 (*NRF2*) targets genes that can suppress lipid peroxidation and the accumulation of free iron, so NRF2 significantly transcriptionally regulates the expression of antiferroptotic genes [44]. Activation of the *NRF2* pathway can lead to a rapid increase in ROS, which can promote ferroptosis. Chang et al. determined that dipeptidyl peptidase 9 (*DPP9*) could bind to *KEAP1* via a conserved ESGE motif and then regulate the *KEAP1/NRF2/SLC7A11* axis, resulting in the suppression of ferroptosis [45]. Ni et al. demonstrated that disulfiram/copper treatment prolonged the half-life of *NRF2*, reducing its degradation and reducing the expression of nuclear protein localization homolog 4 (*NPL4*), which is a ubiquitin protein-proteasome system involved in *NRF2* degradation, ultimately leading to oxidative stress and ferroptosis. However, it is noteworthy that surmounting the compensatory increase in *NRF2* triggered by *NPL4* inhibition amplifies disulfiram/copper-induced oxidative stress and ferroptosis in RCC [46].

#### *HIF-2α* signaling pathway

Unlike HIF-1α, HIF-2α functions as an oncogenic factor to promote the progression of RCC [47]. Yang et al. reported that O-GlcNAc transferase (*OGT*) increased the expression of *HIF-2α* by repressing degradation mediated through the ubiquitin‒proteasome system. Furthermore, the *OGT/HIF-2α* axis increases the sensitivity of ccRCC to ferroptosis [48].

#### Mitochondrial metabolism

Mitochondria are sites of ROS production, and excess ROS can trigger ferroptosis via the Fenton reaction. Yang et al. demonstrated that succinate dehydrogenase (*SDH*) inhibition dampened oxidative phosphorylation, which was characterized by decreased mitochondrial ROS levels, decreased cellular ROS and decreased peroxide accumulation, thus reducing ferroptotic events and restoring ferroptotic cell death [49].

### Molecular mechanism of ferroptosis in PCa

**Fig. 3:**
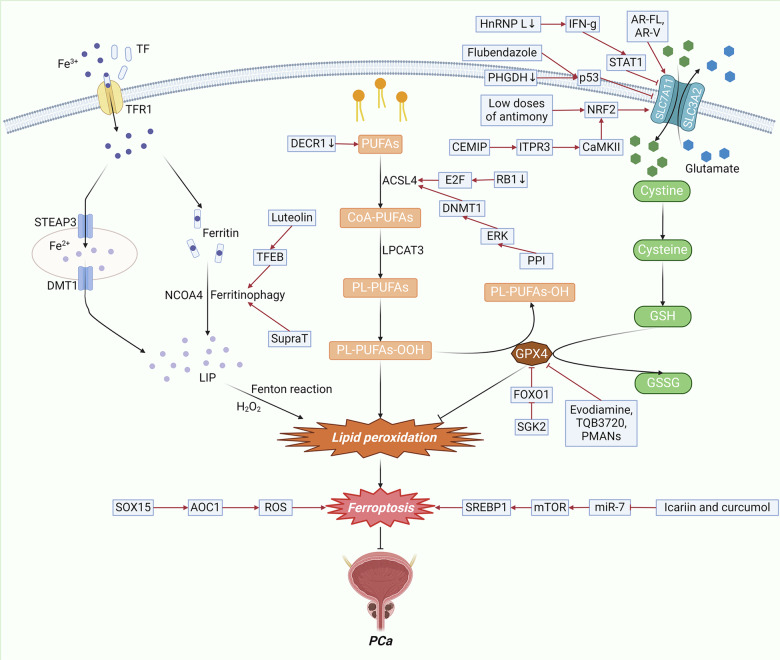
The molecular mechanism of ferroptosis in PCa. In PCa, ferroptosis can be regulated through mechanisms involving GSH, lipid metabolism, iron metabolism, the ROS signaling pathway, and the *mTOR* signaling pathway. Abbreviations: PUFAs, polyunsaturated fatty acids; CoA, coenzyme A; PL-PUFAs, phospholipid-containing PUFAs; *ACSL4*, acyl-CoA synthetase long-chain family member 4; *LPCAT3*, lysophosphatidylcholine acyltransferase 3; *TF*, transferrin; TFR1, transferrin receptor 1; *STEAP3*, iron oxide reductase steam 3; *DMT1*, divalent metal transporter 1; *NCOA4*, nuclear receptor coactivator 4; *LIP*, labile iron pool; *SLC7A11*, solute carrier family 7 member 11; *SLC3A2*, solute carrier family 3 member 2; *GSH*, glutathione; *GSSG*, Oxidized glutathione; *GPX4*, glutathione peroxidase 4; *NRF2*, nuclear factor erythroid 2-related factor 2; ROS, Reactive Oxygen Species; *SGK2*, serum/glucocorticoid regulated kinase 2; *CEMIP*, cell migration-inducing protein; *HnRNP L*, heterogeneous nuclear ribonucleoprotein L; *PHGDH*, phosphoglycerate dehydrogenase; *PPI*, Polyphyllin I; *TFEB*, transcription factor EB; *AOC1*, amine oxidase copper-containing 1.

Ferroptosis can be regulated by GSH, lipids, iron, and mitochondrial metabolism in PCa (Fig. 3).

#### *GPX4/GSH* steady-state system

PCa can also be regulated by ferroptosis through the *GPX4/GSH* steady-state system. Shi et al. showed that exposure to low doses of antimony promotes cell proliferation in PCa, which is attributed to the inhibition of ferroptosis via the activation of the *NRF2/SLC7A11/GPX4* pathway [50]. Zhou et al. revealed that flubendazole induced the expression of P53, which partly accounted for the inhibition of *SLC7A11* transcription, and subsequently downregulated *GPX4*, which is a major antiferroptosis gene [51]. Zhang et al. reported that the lncRNA *OIP5-AS1* promoted the progression of PCa and conferred resistance to ferroptosis through the *miR-128-3p/SLC7A11* signaling pathway [52]. Cell migration-inducing protein (*CEMIP*) is an intranuclear protein that is abnormally highly expressed in PCa and promotes tumor invasion and migration [53]. Liu et al. reported that *CEMIP* inhibited ferroptosis through the *ITPR3/CaMKII/NRF2/SLC7A11* pathway [54]. Zhou et al. demonstrated that knockdown of heterogeneous nuclear ribonucleoprotein L (*HnRNP L*) resulted in increased production of interferon gamma (*IFN-γ*), which induced ferroptosis in castration-resistant prostate cancer (CRPC) cells through the *STAT1/SLC7A11/GPX4* signaling axis [55]. Wang et al. reported that the inhibition of phosphoglycerate dehydrogenase (*PHGDH*) induced ferroptosis by reducing *GSH/GSSG* levels, elevating LipROS production and suppressing *SLC7A11* expression through the activation of the p53 signaling pathway [56]. Ferroptosis can also be regulated by direct action downstream of *GPX4*. Cheng et al. reported that the overexpression of serum/glucocorticoid regulated kinase 2 (*SGK2*) facilitated the translocation of *FOXO1* from the nucleus to the cytoplasm, alleviating the inhibitory effect of *FOXO1* on *GPX4* and consequently inhibiting ferroptosis [57]. Yu et al. reported that evodiamine, a natural alkaloid compound derived from the fruit of Evodiae fructus, can induce ferroptosis by decreasing the expression of *GPX4* [58]. Zhang et al. discovered that TQB3720, a second-generation androgen receptor (*AR*) antagonist, promoted ferroptosis in PCa cells by diminishing the binding of the *AR* and specificity protein 1 (*SP1*) transcriptional complex to the *GPX4* promoter [59]. In addition, Wang et al. engineered an inorganic metal-free nanoplatform, namely, PSMA-targeted arsenic nanosheets (PMANs), which can enhance GSH consumption, suppress the expression of *SLC7A11* and *GPX4*, and promote the generation of ROS and lipid peroxides (LPOs). These concerted actions synergistically contribute to the promotion of ferroptosis in PCa cells [60].

#### Lipid metabolism

PCa can also participate in the regulation of ferroptosis through lipid metabolism. Wang et al. demonstrated that loss of the tumor suppressor gene *RB1* coupled with transcriptional family *E2F* activation sensitizes PCa cells to ferroptosis. This sensitivity is attributed to the upregulation of *ACSL4* expression and the enrichment of *ACSL4*-dependent arachidonic acid–containing phospholipids, which are crucial components of ferroptosis execution [61]. Sun et al. demonstrated that acute treatment with the antiandrogen enzalutamide led to a reduction in GSH production and an increase in lipid peroxidation, lending to the induction of ferroptosis in PCa cells. Specifically, the cystine transporter gene *SLC7A11* has been identified as a pivotal *AR* target. Full-length *AR* (AR-FL) was observed to transactivate *SLC7A11* transcription by directly occupying the *SLC7A11* promoter and putative enhancer regions. *AR* variants (AR-Vs) preferentially bind to the *SLC7A11* enhancer and upregulate *SLC7A11* expression, thereby conferring resistance to ferroptosis induced by ENZ treatment. Therefore, *SLC7A11* acts as a direct target gene for both AR-FL and AR-Vs [62]. Nassar et al. discovered that the knockdown of *DECR1*, a negatively regulated AR target gene, resulted in the cellular accumulation of PUFAs, heightened mitochondrial oxidative stress, and increased lipid peroxidation, ultimately leading to the induction of ferroptosis [63]. Zou et al. reported that Polyphyllin I (*PPI*), a steroidal saponin in *Paris polyphylla*, could serve as a ferroptosis inducer to promote ferroptosis in CRPC cells through the *ERK/DNMT1/ACSL4* axis [64].

#### Iron metabolism

PCa may also be involved in the regulation of ferroptosis through lipid metabolism. Kumar et al. reported that supraphysiological levels of testosterone (SupraT) initiate ferroptosis by inducing two parallel autophagy-mediated processes, specifically ferritinophagy and nucleophagy [65]. Similarly, Fu et al. reported that luteolin induces ferroptosis in PCa cells by increasing autophagy and ferritinophagy through the promotion of transcription factor EB (*TFEB*) nuclear translocation [66].

#### Mitochondrial metabolism

PCa may also play a role in the regulation of ferroptosis through mitochondrial metabolism. Ding et al. showed that the anticancer effect of amine oxidase copper-containing 1 (*AOC1*) is mediated via its impact on spermidine, leading to the activation of ROS and subsequent induction of ferroptosis. The expression of *AOC1* in PCa was positively regulated by the transcription factor *SOX15*. Therefore, *SOX15* can induce ferroptosis in PCa cells through the *SOX15/AOC1/ROS* axis [67].

### Molecular mechanism of ferroptosis in BCa

**Fig. 4:**
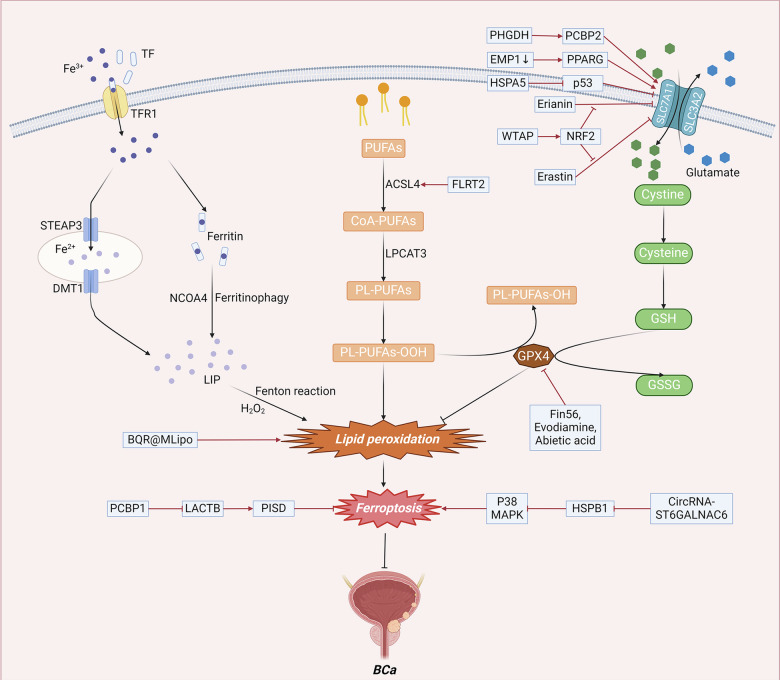
The molecular mechanism of ferroptosis in BCa. In BCa, ferroptosis can be regulated through various mechanisms, including GSH levels, lipid metabolism, mitochondrial metabolism, and the *MAPK* signaling pathway. Abbreviations: PUFAs, polyunsaturated fatty acids; CoA, coenzyme A; PL-PUFAs, phospholipid-containing PUFAs; *ACSL4*, acyl-CoA synthetase long-chain family member 4; *LPCAT3*, lysophosphatidylcholine acyltransferase 3; *TF*, transferrin; *TFR1*, transferrin receptor 1; *STEAP3*, iron oxide reductase steam 3; *DMT1*, divalent metal transporter 1; *NCOA4*, nuclear receptor coactivator 4; LIP, labile iron pool; *SLC7A11*, solute carrier family 7 member 11; *SLC3A2*, solute carrier family 3 member 2; GSH, glutathione; *GSSG*, Oxidized glutathione; *GPX4*, glutathione peroxidase 4; *NRF2*, nuclear factor erythroid 2-related factor 2; ROS, Reactive Oxygen Species; *PHGDH*, phosphoglycerol dehydrogenase; *EMP1*, epithelial membrane protein 1; *FLRT2*, fibronectin leucine rich transmembrane protein 2; *PCBP1*, poly C binding protein 1.

Ferroptosis in BCa can be regulated through GSH, lipid metabolism, mitochondrial metabolism, and the NRF2 signaling pathway (Fig. 4).

#### *GPX4/GSH* steady-state system

The *GPX4/GSH* steady-state system can also be involved in the regulation of ferroptosis in BCa. *PHGDH*, a key enzyme in the serine synthesis pathway, is highly expressed in numerous cancers [68]. *PHGDH* knockdown decreased *SLC7A11* expression and subsequently induced ferroptosis in BCa cells. Mechanistically, *PHGDH* was observed to bind to *PCBP2*, an RNA-binding protein, and inhibit its ubiquitination-mediated degradation. Consequently, *PCBP2* in turn enhances *SLC7A11* mRNA stability and increases its expression [69]. Liu et al. demonstrated that epithelial membrane protein 1 (*EMP1*) deficiency enhanced tumor metastasis by increasing the expression of *PPARG*, a transcription factor, and promoting its activation, resulting in the upregulation of *pFAK* (Y397) and *SLC7A11*, which are involved in cell migration and antiferroptosis, respectively [70]. Sun et al. determined that *Fin56*, a type 3 ferroptosis inducer, could promote ferroptosis and autophagy in BCa cells. Notably, the induction of ferroptosis by *Fin56* was found to mechanistically depend on the autophagy-mediated degradation of *GPX4* [71]. The classical tumor suppressor gene *p53* can also regulate the expression of *SLC7A11* to promote ferroptosis. Li et al. reported that *p53* activation stimulated the lipoxygenase activity of *ALOX15B* by inhibiting *SLC7A11* to induce ferroptosis in BCa cells, which provided insight into the molecular mechanism underlying the occurrence and development of BCa [72]. Wang et al. reported that heat shock protein family A (*HSP70*) member 5 (*HSPA5*) can inhibit ferroptosis through the *p53/SLC7A11/GPX4* pathway [73].

#### Lipid metabolism

Lipid metabolism can also play a role in the regulation of ferroptosis in BCa. Ding et al. engineered a mitochondrial-targeted liposome loaded with brequinar (BQR) (BQR@MLipo), which was designed to selectively accumulate in mitochondria and inactivate dihydroorotate dehydrogenase (DHODH), resulting in extensive mitochondrial lipid peroxidation and the generation of ROS, ultimately leading to ferroptosis of BCa cells [74]. Fibronectin leucine-rich transmembrane protein 2 (*FLRT2*) is overexpressed in advanced colorectal cancer and is negatively correlated with patient survival [75]. Jiang et al. discovered that *FLRT2* upregulated the expression of *ACSL4*, leading to increased lipid peroxidation and subsequently promoting ferroptosis in human BCa cells [76].

#### Mitochondrial metabolism

The metabolism of mitochondria may also play a role in the regulation of ferroptosis in BCa. Luo et al. reported that poly C binding protein 1 (*PCBP1*) protected BCa cells against mitochondrial injury and ferroptosis through the *LACTB/PISD* axis. Mechanistic insights revealed that *LACTB* mRNA is a novel transcript that can bind to *PCBP1*. The upregulation of *LACTB* facilitated erastin-induced ferroptosis and induced mitochondrial dysfunction. Moreover, the overexpression of *LACTB* reversed the ferroptosis protection mediated by *PCBP*-1, including a decrease in ROS and an enhancement of mitochondrial function [77]. Xu et al. reported that abietic acid (AA)-induced ferroptosis in BCa cells was mediated by a combination of multiple factors. This included the elevation of ROS, intracellular iron, and MDA. Furthermore, AA treatment suppressed *GPX4* and enhanced *HO-1* [78].

#### *NRF2* signaling pathway

The *NRF2* signaling pathway can also play a role in the regulation of ferroptosis in BCa. Xiang et al. showed that erianin facilitated the accumulation of lethal lipid-based ROS and the depletion of *GSH*, inducing ferroptosis. Mechanistically, *NRF2* served as a key determinant of erianin-triggered ferroptosis. The activation of *NRF2* through *TBHQ* treatment protected against erianin-induced ferroptosis and elevated the expression of *GPX4*, ferritin, xCT and glutaminase. On the other hand, *NRF2* knockdown increased the activity of ROS and MDA, decreased GSH levels and decreased the expression of negative regulatory proteins associated with ferroptosis [79]. Wang et al. revealed that the m6A methyltransferase *WTAP* could methylate the 3’-UTR of the RNA of the endogenous antioxidant factor *NRF2*, consequently increasing the mRNA stability of *NRF2* and thus inhibiting erastin-induced ferroptosis [80].

## The role of ferroptosis in the diagnosis of urological malignancies

Patients diagnosed with advanced urological malignancies often have a bleak prognosis and a heightened risk of mortality [81, 82]. Therefore, identifying ferroptosis-related potential prognostic markers is important for the diagnosis and treatment of urological malignancies (Table 2).

**Table 2:** The roles of ferroptosis in the diagnosis of urological malignancies.

Cancers	Target	Relationship with ferroptosis	Biological function	Reference
RCC	CCR4,NR3C2	Ferroptosis and immune-related differentially expressed genes	Better OS	[84]
	CMTM3, IFITM1 and MX2	Ferroptosis and immune-related differentially expressed genes	Worse OS	[84]
	CRFGs	Cuproptosis-related Ferroptosis genes	Worse OS	[85]
	CBS、CD44、AKR1C2、CHAC1 and SLC7A11	Ferroptosis-related gene	Worse OS	[86]
	HMOX1 and HMGCR	Ferroptosis-related gene	Better OS	[86]
	SLC7A11	Ferroptosis-related gene	Worse OS	[89]
PCa	ARPC1A	Inhibit	Worse DFS	[93]
	E2F1, CDC20, TYMS,and NUP85	Ferroptosis-related gene prognostic index	Worse DFS	[94]
	five frlncRNAs	Ferroptosis-related lncRNA	Higher disease grades and a greater number of infiltrating immune cells	[95]

### RCC

Sixty percent of individuals with RCC are asymptomatic, resulting in late detection of many cancers; more than 25% exhibit evidence of metastasis at the time of diagnosis [83]. Therefore, the development of diagnostic and prognostic markers for RCC is imperative. Xing et al. proposed that five ferroptosis-induced differentially expressed genes (FI-DEGs), namely, *CCR4*, *CMTM3*, *IFITM1*, *MX2*, and *NR3C2*, may serve as valuable prognostic and diagnostic biomarkers for patients diagnosed with RCC [84]. Luo et al. combined cuproptosis and ferroptosis and screened six cuproptosis-related ferroptosis genes (CRFGs) that showed promise in predicting the prognosis and therapeutic outcome of patients with RCC [85]. Sun et al. constructed a 7-gene ferroptosis-related prognostic signature by analyzing a panel of ferroptosis-related genes to predict overall survival (OS) in RCC patients [86].

Immunotherapy is a therapeutic strategy that combats malignant tumors by activating or enhancing the body’s immune system to recognize and destroy cancer cells [87]. Although immunotherapy has been applied to a variety of tumors, including urological malignancies, its clinical efficacy remains suboptimal in some patients. The immune system plays a critical role in tumor immunosurveillance through the infiltration of adaptive and innate immune cells into the tumor microenvironment (TME), where they regulate tumor progression. Thus, a comprehensive understanding of immune infiltration within the TME is essential for improving response rates and developing novel immunotherapeutic strategies [88]. The interplay between ferroptosis and tumor immune infiltration is a critical area that warrants further investigation. Tumor immune infiltration, involving various immune cells such as T cells, macrophages, and dendritic cells, plays a pivotal role in shaping the tumor microenvironment and influencing tumor growth and metastasis. Emerging evidence suggests that ferroptosis not only induces cancer cell death but also modulates the immune landscape within tumors. Ferroptotic cancer cells can release damage-associated molecular patterns (DAMPs) and other immunogenic signals that activate and attract immune cells to the tumor site. This process can enhance antitumor immunity by promoting the recruitment and activation of cytotoxic T cells and macrophages, which are essential for attacking and eliminating tumor cells. However, ferroptosis can also lead to the release of lipid peroxidation products, which may have immunosuppressive effects, creating a complex interplay between ferroptosis and immune responses. The study by Xu et al. found that the overexpression of SLC7A11 was associated with poor prognosis in patients with ccRCC and that SLC7A11 expression was positively correlated with the infiltration of immune cells and their corresponding markers, including CD8^+^ T cells and myeloid dendritic cells. Thus, SLC7A11 could serve as a potential prognostic biomarker for ccRCC and as an indicator of immune cell infiltration within tumors [89]. The study by Zong et al. identified eight ferroptosis-associated lncRNAs in ccRCC that could be used to predict overall patient survival. These lncRNAs were also correlated with the immune microenvironment, immunotherapeutic response, and drug sensitivity in ccRCC, offering a potential method for assessing immunotherapeutic efficacy in ccRCC patients [90].

### PCa

Although prostate-specific antigen (PSA) has emerged as the principal screening tool for PCa in clinical settings, it exhibits low specificity and sensitivity [91, 92]. Ji et al. suggested that decreased *ARPC1A* expression inhibits PCa cell viability and invasion through ferroptosis. The level of *ARPC1A* may serve as an independent indicator of prognosis in patients with PCa [93]. The ferroptosis-related gene prognostic index (*FRGPI*), constructed using the genes *E2F1*, *CDC20*, *TYMS*, and *NUP85*, has been shown to predict disease-free survival (DFS) in patients with PCa [94]. Liu et al. developed a model based on five ferroptosis-related lncRNAs to predict biochemical recurrence in PCa patients [95].

Liu et al. propose a novel strategy for assessing biochemical recurrence in PCa patients based on five ferroptosis-associated lncRNAs. Moreover, they found that the disease severity in the high-risk group identified by this predictive model was positively correlated with the number of infiltrating immune cells. Additionally, these five lncRNAs were linked to key immune checkpoints [95].

### BCa

Although the five-year survival rate is high for patients diagnosed with BCa at an early disease stage, survival substantially decreases in patients with muscle-invasive or metastatic disease [96]. Therefore, early detection of their prognosis is of paramount importance. Liu et al. identified five ferroptosis-related genes (FRGs) that demonstrate significant potential in stratifying patients with BCa based on their prognosis [97]. Hou et al. discovered a frlncRNA signature that predicts the prognosis of patients with bladder cancer [98].

Li et al. identified 12 lncRNA pairs linked to immune responses and ferroptosis to develop a risk prediction model for BCa patients. Notably, the high-risk group exhibited significantly worse OS and demonstrated a positive correlation with the majority of tumor-infiltrating immune cells [99].

## The roles of ferroptosis in the occurrence and treatment of urological malignancies

Ferroptosis induction has been shown to effectively inhibit tumor growth and metastasis, so ferroptosis has considerable potential for application in the treatment of urological tumors. Recent relevant studies are listed in Table 3.

**Table 3:** The roles of ferroptosis in the treatment of urological malignancies.

Cancer	Drug	Target	Ferroptosis (inducer inhibitor)	Biological function	Model	Reference
RCC		KLF2	Induce	Suppress proliferation, migration and invasion abilities of RCC cells	In vitro and in vivo	[29]
		AIM2	Inhibit	Promote RCC progression and sunitinib resistance	In vitro and in vivo	[39]
		MLYCD	Induce	Reduce tumor growth and reverse resistance to sunitinib	In vitro and in vivo	[43]
		OGT	Induce	Promote the proliferation, clone formation, and invasion of VHL-mutated ccRCC cells	In vitro and in vivo	[48]
		Knockdown of SETD2	Induce	Promote tumor cell death	In vitro and in vivo	[103]
		STEAP3	Inhibit	Poor survival and prognosis	In vitro	[104]
	RSL3 or Erastin		Induce	Overcome resistance to everolimus	In vitro	[105]
	MIL-101(Fe)@RSL3		Induce		In vitro and in vivo	[107]
		NCOA4	Induce	Maintain ferritinophagy	In vitro and in vivo	[108, 109]
		CX3CL1	Induce	Inhibit tumor cell proliferation and metastasis	In vitro and in vivo	[110]
PCa	Flubendazole		Induce	Inhibit cell proliferation,cause cell cycle arrest in G2/M phase and promoted cell death	In vitro and in vivo	[51]
		PHGDH	Inhibit	Promote cell growth and Enza resistance in CRPC cells	In vitro and in vivo	[56]
	TQB3720		Induce	Inhibit the growth of prostate cancer	In vitro and in vivo	[59]
		AOC1	Induce	Reduce proliferation and migration in prostate cancer	In vitro and in vivo	[67]
	Erastin or RSL3		Induce	Halt prostate cancer cell growth and migration in vitro and tumor growth in vivo	In vitro and in vivo	[111]
	Icariin and curcumol		Induce	Induce autophagy in PCa cells	In vitro and in vivo	[113]
	Ferumoxytol		Induce	Enhance NK cells’ function	In vitro and in vivo	[114]
BCa	NCT-502	PHGDH	Inhibit	Induce tumor progression	In vitro and in vivo	[69]
		EMP1	Induce	Suppress tumor cell metastasis	In vitro and in vivo	[70]
	BQR@MLipo		Induce	Trigger ferroptosis of bladder cancer	In vitro and in vivo	[74]
		FLRT2	Induce	Suppress tumor cell growth, migration and invasion	In vitro	[76]
	Abietic acid (AA)		Induce	Selectively inhibited the viability of BC cells	In vitro and in vivo	[78]
		RP11-89	Inhibit	Promote cell proliferation, migration and tumorigenesis and inhibited cell cycle arrest	In vitro and in vivo	[117]
		LUCAT1	Inhibit	Foster cell proliferation, migration, and invasion	In vitro and in vivo	[119]
	Evodiamine		Induce	Suppress the migratory ability, decreas the expression of mesenchymal markers, and increase epithelial marker expression	In vitro and in vivo	[120]

### RCC

As mentioned, ferroptosis is widely believed to play a regulatory role in the progression of RCC [100]. Accordingly, directing therapeutic directions toward regulators associated with ferroptosis has emerged as a highly promising strategy for addressing this disease [101].

SET domain-containing 2 (*SETD2*) plays a significant role as an epigenetic regulator and has a high mutation rate in RCC [103]. Xue et al. reported that suppression of the epigenetic molecule *SETD2* markedly increased the sensitivity of cells to ferroptosis inducers, which facilitates tumor cell death, indicating that *SETD2* may be a promising therapeutic target for treating ccRCC [104]. Ye et al. reported that RCC cell lines in which *STEAP3* expression was knocked down exhibited increased sensitivity to ferroptosis, and this effect was attributed to the *p53/xCT* pathway. Therefore, *STEAP3* has the potential to become a new biomarker and target for the treatment of RCC [105].

Chemoresistance is a major obstacle in the treatment of RCC and leads to poor prognosis. Recent investigations have illuminated the potential of ferroptosis inducers in overcoming sunitinib resistance. Wang et al. discovered that *AIM2* serves as a novel biomarker for RCC and plays a role in promoting both RCC progression and resistance to sunitinib through an inflammasome-independent mechanism [39]. Therefore, this could provide a new therapeutic target for RCC diagnosis and inversion of sunitinib resistance. The ferroptosis inducers *RSL3* and erastin may synergize with everolimus by inhibiting the *mTOR/4EBP1* axis. This combination strategy holds potential for overcoming resistance to everolimus [106].

However, existing ferroptosis-inducing therapies are limited by a lack of precise targeting. Therefore, it is conceivable to enhance their efficacy by integrating them with targeted nanomedicine delivery systems [107]. Ni et al. designed iron-based metal-organic framework nanoparticles that can deliver RSL3 (MIL-101(Fe)@RSL3) in a targeted manner to ccRCC cells. As a pH-responsive nanodrug, MIL-101(Fe)@RSL3 induces cellular iron overload and promotes hydroxyl radical (•OH) generation via the Fenton reaction. This mechanism targets polyunsaturated fatty acids (PUFAs), resulting in the anomalous accumulation of lipid peroxides (L-OOH). Furthermore, RSL3 directly inhibits *GPX4* to detoxify L-OOH. Concurrently, ferrous ions further catalyze the irreversible conversion of highly reactive lipid alkoxyl radicals (L-O•) from L-OOH, initiating a waterfall-like cascade leading to ferroptosis [108].

Phosphorylated NCOA4 has been found to promote ferroptosis by maintaining ferritinophagy [109]. And in ccRCC, NCOA4 deficiency led to impaired immune cell infiltration through the disruption of IFN-γ receptor signaling, which was associated with disease progression and poor prognosis [110]. Therefore, targeting NCOA4 may represent a promising therapeutic strategy that combines ferroptosis induction with immunotherapy. Gong et al. reported that overexpression of the chemokine *CX3CL1* inhibited tumor cell proliferation and metastasis by enhancing tumor sensitivity to ferroptosis in ccRCC. Moreover, as the expression level of CX3CL1 is closely correlated with the infiltration of CD8+ T cells in the TME, *CX3CL1* can also act as a promising predictor of immunotherapy outcomes in ccRCC patients in the clinic [102].

### PCa

Recent studies have demonstrated that treatment-resistant PCa cells exhibit sensitivity to two ferroptosis inducers, erastin and *RSL3*. Specifically, erastin and *RSL3* were effective at inhibiting PCa cell growth, as well as migration in vitro and tumor growth in vivo [111, 112]. These results indicate that the induction of ferroptosis may have the potential to become a new method for treating PCa. As mentioned earlier, *SOX15* has the potential to promote ferroptosis in PCa cells through the *SOX15/AOC1/ROS* axis. Moreover, elevated expression of *AOC1* is strongly associated with diminished proliferation and migration in PCa. Therefore, targeting *AOC1* and *SOX15* is a promising approach for the treatment of PCa [67].

In addition to the above targets, numerous compounds that can inhibit PCa progression by regulating ferroptosis in PCa cells have been identified in recent studies. For example, Xu et al. reported that icariin (*ICA*), a flavonoid compound isolated from the traditional Chinese medicine Epimedium, and curcumol, a sesquiterpene compound, synergistically regulated the *miR-7/mTOR/SREBP1* pathway, inducing ferroptosis and subsequently triggering autophagy in PCa cells [113]. Kim et al. reported that the cytotoxic function of natural killer (NK) cells could be enhanced by ferroptosis, a clinical-grade iron oxide nanoparticle, in cancer cells mediated by ferumoxytol combined with NK cells, which is beneficial for the death of PCa cells [114].

The anti-androgenic drug enzalutamide, which is currently in clinical use, is also associated with ferroptosis. Wang et al. reported a link between high expression of *PHGDH* and enzalutamide resistance in CRPC cells. Additionally, increased *PHGDH* levels were found to account for ferroptosis resistance by maintaining redox homeostasis in enzalutamide-resistant CRPC cells, ultimately promoting cell growth and enzalutamide resistance in CRPC cells. Furthermore, the pharmacological inhibition of *PHGDH* using NCT-503 effectively inhibited cell growth, induced ferroptosis, and overcame enzalutamide resistance in ENZ-resistant CRPC cells. Therefore, the combination of ferroptosis inducers and targeted inhibition of *PHGDH* has emerged as a promising therapeutic strategy for overcoming enzalutamide resistance in CRPC [56].

The immunotherapeutic drug BEBT-908, a dual inhibitor of PI3K/HDAC, inhibited tumor cell growth by inducing ferroptosis in cancer cells. Mechanistically, this process led to the upregulation of MHC class I and the activation of endogenous IFNγ signaling within the cells [115]. Additionally, inhibiting autophagy could trigger anti-tumor immune memory by increasing MHC-I expression in PCa cells, particularly when combined with PD-L1 blockade, thereby exerting synergistic anti-PCa effects [116]. These findings underscore the potential of leveraging ferroptosis for synergistic immunotherapy in PCa. NK cell-based immunotherapy is a promising therapeutic approach. In combination with ferroptosis, mediated by ferumoxytol-an iron oxide nanoparticle—NK cell therapy has shown enhanced efficacy. This combination treatment leads to the upregulation of ULBPs, ligands for the NK cell-activating receptor NKG2D, as well as increased expression of HMGB1 and PD-L1 in cancer cells. In in vivo experiments, the combination of ferumoxytol-mediated ferroptosis and NK cell therapy resulted in a significant reduction in tumor volume. Thus, ferumoxytol-mediated ferroptosis combined with NK cell therapy demonstrates potential synergistic anticancer effects [114].

### BCa

Luo et al. reported that the long noncoding RNA (lncRNA) *RP11-89* can enhance cell proliferation, migration and tumorigenesis while inhibiting cell cycle arrest through the *miR-129-5p/PROM2* axis. *RP11-89* may serve as a prospective biomarker for targeted therapy in BCa [117]. Jiang et al. reported decreased *FLRT2* expression in human BCa patients and that increased *FLRT2* expression correlated with a decreased survival rate. Functional investigations indicated that downregulation of *FLRT2* facilitated tumor cell growth, migration, and invasion, suggesting that *FLRT2* could be a tumor suppressor gene [76]. The lncRNA lung cancer-associated transcriptome 1 (*LUCAT1*) is abnormally expressed in various tumor tissues and is closely related to the proliferation and invasion of tumor cells [118]. Cao et al. revealed that *LUCAT1* promoted cell proliferation, migration, and invasion while simultaneously inhibiting ferroptosis in BCa. These findings suggested that *LUCAT1* could serve as a promising therapeutic target for BCa [119].

Evodiamine (*EVO*), a quinazoline alkaloid, has been shown to exert anticancer effects by inhibiting cell proliferation and tumor growth. *EVO* also functions as a novel inducer that can activate ferroptosis in BCa cells, showing its potential as a therapeutic agent for BCa [120]. Kong N et al. showed that baicalin, a medicinal plant, exerts its anticancer effects by inducing ferroptosis in BCa cells [121].

Checkpoint blockade immunotherapy (CBI) has demonstrated remarkable benefits in cancer therapy [122]. However, the low responsiveness of CBI has hindered its application in the treatment of BCa [122, 123]. Ferroptosis, which has the potential to induce immunogenic cell death, can enhance the responsiveness of CBI [124, 125]. Ding Q et al. developed a mitochondrial-targeted liposome loaded with brequinar (BQR) (BQR@MLipo) to enhance mitochondria-related ferroptosis in situ in BCa. BQR@MLipo not only induces ferroptosis through multiple factors, as mentioned earlier but also significantly accumulates in bladder tumors and successfully initiates the infiltration of CD8^+^ T cells into the TME, enabling efficient CBI to inhibit bladder tumor growth [74].

### Clinical challenges in targeting ferroptosis with therapeutic agents

Targeting ferroptosis presents a promising anti-cancer strategy. However, the clinical translation of ferroptosis-inducing agents faces several significant challenges that require further research to overcome for practical application. Firstly, the activation and regulatory mechanisms of ferroptosis within the body are highly complex and interconnected. Tumorigenesis is a multifaceted process involving metabolic disturbances, where ferroptosis can lead to the death or suppression of tumor cells but, in certain contexts, may paradoxically promote tumor formation [126, 127]. Thus, further research is essential to elucidate the precise mechanisms underlying ferroptosis. Secondly, ferroptosis may exhibit potential synergistic effects with other forms of cell death, such as apoptosis [128] and autophagy [129]. Consequently, more in-depth studies are warranted to elucidate the relationships between ferroptosis and these common cell death pathways. Understanding these correlations is crucial for mitigating potential interferences from other cell death mechanisms and for enhancing the therapeutic efficacy of ferroptosis-based treatments. Thirdly, there is a significant lack of effective and specific drugs capable of safely inducing ferroptosis in cancer cells [126]. Current ferroptosis-inducing agents are hindered by poor targeting and low cellular uptake, leading to potential toxic side effects [7]. As previously noted, ongoing studies aim to enhance drug targeting and minimize side effects by designing targeted nanodelivery systems to encapsulate ferroptosis agonists or inhibitors [74, 108]. Compared to conventional drugs, nanodelivery systems offer superior biocompatibility and targeting capabilities, which reduce toxic side effects while enhancing the stability and bioavailability of the encapsulated agents [130]. Additionally, the development of combination therapeutic strategies based on ferroptosis presents a viable approach to mitigating toxicity. For instance, combining cisplatin with the ferroptosis agonist erastin has demonstrated significant synergistic effects in enhancing anti-tumor activity in lung and colon cancers [131, 132].

To address these challenges, future research should focus on several key issues. First, elucidating the molecular mechanisms underlying ferroptosis pathways, particularly those associated with tumor specificity, to improve the targeting precision of therapeutic agents. Second, developing combination therapy strategies, where ferroptosis inducers are used alongside other anticancer drugs or treatment modalities (e.g., radiotherapy, immunotherapy), may enhance therapeutic efficacy while minimizing adverse effects. Additionally, the design of clinical trials should prioritize personalized treatment approaches, tailoring the therapeutic regimen based on the patient’s genotype and tumor characteristics. Although the clinical application of ferroptosis-related drugs in urological malignancies presents significant challenges, ongoing research into the underlying mechanisms and the development of diverse therapeutic strategies hold promise for establishing ferroptosis as a viable and effective cancer treatment option.

## Discussion and perspectives

Ferroptosis, a novel form of PCD that differs from apoptosis and necrosis, plays a pivotal role in the growth inhibition of various tumors and influences the tumor immune microenvironment [21, 133]. Ferroptosis has emerged as a significant factor in tumor suppression and represents a therapeutic target in various cancers, including non-small cell lung cancer, liver cancer, pancreatic cancer, and breast cancer. A notable array of iron death inducers has been developed as prospective cancer treatment modalities. For instance, in triple-negative breast cancer, Compound C18 has been shown to impede tumor cell activity by promoting iron death in tumor cells [134]. The fusion of ferroptosis inducers with nanomaterials facilitates targeted delivery, controlled release, biocompatibility, and minimal toxicity, thereby expanding their potential applications [135]. T cells play a role in promoting ferroptosis in tumor cells, suggesting a novel antitumor mechanism. Consequently, the integration of ferroptosis with tumor immunotherapy holds promise as an innovative approach for cancer treatment [127].

Due to its significant role in regulating the biological functions of tumor cells, ferroptosis has also emerged as a novel avenue for exploring treatments for urologic malignancies and advancing drug development in recent years [101, 136, 137]. In terms of urological tumors, investigators have screened numerous targets, including target genes [138, 139], miRNAs [140], and circRNAs [141], which exhibit the capacity to regulate ferroptosis in tumor cells by modulating pivotal pathways, including GSH metabolism, iron metabolism, lipid metabolism, and other intricate molecular pathways [142]. In addition to the above targets, recent studies have attempted to apply ferroptosis inducers [111], natural compounds [66] and analogous agents in the investigation of urologic malignancies. Moreover, Ding Q et al. [74] and Ni W et al. [108] addressed the challenges related to inadequate targeting and limited specificity of ferroptosis inducers by integrating them with nanomaterials, thereby broadening the potential for ferroptosis in clinical applications. Furthermore, the induction of ferroptosis in tumor cells may reverse drug resistance in tumor cells, offering innovative ideas for the combination of ferroptosis inducers and chemotherapeutic agents [43, 56].

However, the current study has certain limitations, including heterogeneity, inadequate sample sizes, and the absence of in vivo experiments. Future investigations should prioritize addressing these deficiencies to yield more comprehensive and robust results. Additionally, given that current research on the regulatory mechanisms and biological functions of ferroptosis in the urinary system is in its early stages, despite being a trending research topic in the diagnosis and treatment of urinary tumors, there remains a paucity of corresponding clinical experiments to thoroughly validate its efficacy. Moreover, as a prognostic biomarker, its reliability and reproducibility necessitate further validation through extensive studies involving larger sample sizes.

## Conclusion

In conclusion, we present a comprehensive overview of the mechanisms governing ferroptosis in urologic malignancies and discuss its potential clinical applications. Despite these limitations, delving more deeply into the molecular intricacies of ferroptosis in urologic malignancies holds promise for generating innovative strategies for prevention, identifying therapeutic targets, and establishing robust prognostic biomarkers for these malignancies.
